# Efficacy of beta-blockers in preventing cardiotoxicity in breast cancer patients receiving anthracyclines: a GRADE-assessed systematic review and meta-analysis with trial sequential analysis

**DOI:** 10.3389/fcvm.2026.1926758

**Published:** 2026-10-09

**Authors:** Victor M. Samperio, Lorena M. Murrieta Bruciaga, Jesús Antonio Castañeda Martínez, Vanessa Coronado Porchas, Alejandro Chen Liang, Yeison Cruz Castillo, Aishwarya Ghonge, Aishwarya Saripalli, Thomas Schulz

**Affiliations:** 1 Department of Medicine, Eisenhower Medical Center, Rancho Mirage, CA, United States; 2 Universidad del Valle de México, Hermosillo, Sonora, México; 3 Universidad de Sonora, Hermosillo, Sonora, México; 4 Centro de Estudios Universitarios Xochicalco Campus Tijuana, Tijuana, Baja California, México; 5 Universidad Autónoma de Santo Domingo, Santo Domingo, República Dominicana; 6 Kern Medical Center, Bakersfield, CA, United States; 7 Lucy Curci Cancer Center, Eisenhower Health, Rancho Mirage, CA, United States

**Keywords:** anthracycline (AC), beta-blocker (BB), breast cancer, cardiotoxicity, left ventricular ejecting fraction (LVEF)

## Abstract

**Purpose:**

Anthracycline-induced cardiotoxicity remains an important complication in patients with breast cancer receiving systemic therapy, and effective preventive strategies remain limited. We conducted a systematic review and meta-analysis to evaluate the cardioprotective effects of beta-blockers in anthracycline-treated breast cancer patients.

**Methods:**

Following PRISMA guidelines, Pubmed, Web of Science, CINAHL, and Cochrane were systematically searched, and the protocol was preregistered on PROSPERO (CRD420261366946). The primary outcome was left ventricular ejection fraction (LVEF), while the secondary outcome was E/A ratio. Certainty and robustness of the evidence were assessed using GRADE approach and trial sequential analysis (TSA). A meta-analysis was performed using R version 4.6.0, and heterogeneity was assessed using *I*^2^ statistics.

**Results:**

A total of 8 studies including 772 patients were analyzed. Beta-blocker therapy was associated with significant preservation of LVEF vs. placebo or standard care (MD, 2.85; 95% CI, 0.58–5.12; *p* = 0.02; *I*^2^ 95%). No significant improvement was observed for E/A ratio (MD, 0.10; 95% CI, −0.12 to 0.31; *p* = 0.25; *I*^2^ 69.7%). However, sensitivity analysis resulted in a statistically significant pooled effect (MD = 0.16, 95% CI: 0.03 to 0.29, *p* = 0.01; *I*^2^ = 35.9%). TSA showed that the Z-curve crossed the monitoring boundaries favoring beta-blockers for LVEF; however, the required information size (RIS = 1,785) was not reached.

**Conclusion:**

Beta-blockers were associated with preservation of systolic function in anthracycline-treated breast cancer patients. However, no significant benefit was observed for E/A ratio, and evidence regarding diastolic protection remains inconclusive. Larger, high-quality RCTs are needed to confirm these findings.

**Systematic Review Registration:**

identifier CRD420261366946.

## 1 Introduction

Breast cancer remains one of the most commonly diagnosed malignancies worldwide, with over 2.3 million new cases annually, and continues to represent a leading contributor to cancer-related mortality globally (1). Anthracycline-based chemotherapy has been integral to improving outcomes in early and locally advanced breast cancer; however, as survival improves, treatment-related cardiovascular toxicity has emerged as a determinant of long-term outcomes and a leading cause of non-cancer mortality (2, 3).

Anthracycline-induced cardiotoxicity is mechanistically distinct from other treatment-related cardiovascular toxicities in breast cancer. Proposed mechanisms include oxidative stress, free radical generation, mitochondrial dysfunction, apoptosis, impaired calcium handling, DNA injury, and maladaptive ventricular remodeling (4–10). Because conventional echocardiographic parameters such as left ventricular ejection fraction (LVEF) may remain preserved until advanced myocardial injury occurs, sensitive measures including strain imaging, cardiac biomarkers, and ventricular remodeling parameters have been incorporated for detection of subclinical dysfunction (4–10).

Beta-blockers represent one of the most biologically compelling cardioprotective strategies for anthracycline-induced cardiotoxicity through attenuation of sympathetic activation, reduction in adverse ventricular remodeling, and mitigation of oxidative and apoptotic myocardial injury (4–10). In particular, agents such as carvedilol and nebivolol possess antioxidant and antiapoptotic properties that may counter mechanisms implicated in anthracycline-mediated myocardial damage (4, 6, 8–10). Several randomized studies evaluating carvedilol, nebivolol, and bisoprolol in breast cancer patients receiving anthracycline-based chemotherapy have demonstrated favorable effects on LVEF preservation, strain parameters, ventricular remodeling, diastolic dysfunction, and biomarker release, although the magnitude and consistency of benefit have varied (4–10).

Importantly, prior cardioprotection trials and meta-analyses have evaluated beta-blockers alongside other neurohormonal agents, particularly angiotensin-converting enzyme inhibitors (ACEi) or angiotensin receptor blockers, limiting the ability to isolate the effect of beta-blockade (5). The recently published 2025 SAFE trial provided randomized evidence supporting cardioprotective strategies using bisoprolol and ramipril through a combination design (7). In contrast, several prior meta-analyses combined patients receiving trastuzumab-only regimens and anthracycline-only regimens, which may introduce mechanistic heterogeneity because trastuzumab-associated cardiotoxicity differs biologically from anthracycline-induced myocardial injury (11, 12). This distinction is clinically relevant because trastuzumab-associated cardiotoxicity is mechanistically different from anthracycline-induced myocardial injury, being primarily related to HER2 signaling disruption rather than cumulative oxidative cellular damage. Importantly, all participants across studies included in our analysis received anthracycline-based chemotherapy, although two trials additionally permitted concomitant trastuzumab use in a subset of patients.

Accordingly, we conducted a GRADE-assessed systematic review and meta-analysis with trial sequential analysis (TSA) focused specifically on beta-blockers for the prevention of anthracycline-induced cardiotoxicity in patients with breast cancer. By focusing on anthracycline-treated populations and isolating beta-blocker-based strategies, this analysis aims to provide a more homogenous evaluation of the cardioprotective role of beta-blockade.

## 2 Methodology

This systematic review and meta-analysis followed the Preferred Reporting Items for Systematic Reviews and Meta-analyses (PRISMA) reporting guidelines (13). The protocol was pre-registered at the International Prospective Register of Systematic Review (PROSPERO) with the identifier number CRD420261366946 (14).

### 2.1 Eligibility criteria

#### 2.1.1 Types of study

This review included studies published from inception to August 2026, restricted to those available in English. Eligible study designs included randomized controlled trials and secondary or *post-hoc* analyses of randomized trials when relevant outcome data were available. Excluded studies consisted of cohort studies, case-control studies, editorials, commentaries, case series, dissertations, book chapters, cross-sectional studies, protocol articles, news articles and letters to the editor. After the selected articles were identified, we included additional articles by reference and citation list checking. Studies were excluded if they failed to provide the required information.

#### 2.1.2 Types of participants

This review included participants with breast cancer diagnosed through clinical examination and imaging modalities, including mammography and bilateral breast ultrasound (US), followed by core needle biopsy for histopathological confirmation (15). Participants received anthracycline-based chemotherapy regimens, including doxorubicin, epirubicin, among others. Studies evaluating trastuzumab monotherapy without anthracycline exposure were excluded; however, some included studies permitted concomitant trastuzumab use alongside anthracycline-based chemotherapy.

#### 2.1.3 Types of interventions

This review evaluated the effects of beta-blockers, including carvedilol, nebivolol, and bisoprolol, on the prevention of anthracycline-induced cardiotoxicity. The comparator group consisted of placebo or standard care/control. Exclusion criteria were applied to interventions that were not clearly reported.

#### 2.1.4 Outcomes

The primary outcome of this review included left ventricle ejection fraction (LVEF). Secondary outcomes included the ratio of early to atrial ventricular filling velocities (E/A ratio). Studies were excluded if they did not report data on at least one of the predefined outcomes.

### 2.2 Searching methods

We initially conducted a systematic search on August 2026, on Pubmed, Web of Science, CINAHL, and Cochrane using the following terms: “Breast cancer”, “Anthracyclines”, “Beta blocker” and “Cardiotoxicity”. We have provided all detailed search strategies in the Supplementary Material (Supplementary Tables S1–S4).

#### 2.2.1 Selection of studies

We exported all references to Rayyan (Rayyan Systems Inc., Cambridge, MA, USA), and duplicates were removed (16). Two authors independently (J.A.C.M. and V.C.P.) completed the eligibility assessment, first by title and abstract analysis and subsequently by full-text assessment (J.A.C.M. and V.C.P.). In disagreements between reviewers, a third reviewer (L.M.M.B) helped reach a consensus.

### 2.3 Data extraction

Two reviewers extracted the data (J.A.C.M. and L.M.M.B.), and disagreements were resolved by consensus. When multiple overlapping reports from the same study were identified, the information from the one containing the most relevant information, or the first published report, was included. Extracted data included sample sizes, intervention types, and measured outcomes. Conventional methods were used for data extraction, complemented by specialized tools such as WebPlotDigitizer (Automeris, Austin, TX, USA) for digitizing graphs, Cochrane calculator for statistical conversions, and StatsToDo for advanced calculations (17–19). These outcomes comprised LVEF and E/A ratio. Additional extracted data included subgroup characteristics, such as country of study, risk of bias levels, and study design.

### 2.4 Assessment of risk of bias

To assess the quality of the studies included in the systematic review and meta-analysis, we applied the Cochrane RoB 2.0 tool for randomized controlled trials (RCTs) according to the Cochrane guidelines (20). Two independent reviewers (J.A.C.M. and V.C.P.) evaluated the risk of bias in each study. Any discrepancies were resolved through discussion with a third reviewer (L.M.M.B.).

### 2.5 Assessment of the certainty of the evidence and summary of findings

Two reviewers (L.M.M.B. and A.C.L) independently assessed the overall quality of evidence for each outcome using the five domains of the Grading of Recommendations Assessment, Development, and Evaluation (GRADE) approach study design, risk of bias, inconsistency, indirectness, imprecision, and publication bias, large effect, plausible confounding, dose response gradient, via the GRADEpro GDT software (21, 22). Discrepancies were discussed with a third reviewer (Y.C.C.).

### 2.6 Statistical analysis

We performed a meta-analysis using R version 4.6.0 (R Core Team) with the meta and metafor packages (23, 24). We examined the pooled effect of the outcomes using a random-effects meta-analysis with restricted maximum-likelihood (REML) (25). Hartung-Knapp adjustments were applied for the calculations of random-effects confidence intervals (26). Whenever the number of studies reporting an outcome of interest was insufficient, we only performed a qualitative analysis of the results. We expressed the effect sizes as mean difference (MD) with a 95% confidence interval. We used the *I*^2^ statistic assessed heterogeneity, and the following cut-off values for interpretation: <25%, 25%–50%, and >50% were considered small, medium, and large heterogeneity, respectively (27). For all outcomes, we performed a sensitivity analysis using the leave-one-out method to determine the influence of individual studies on the overall effect (28). To further explore potential sources of heterogeneity, we performed random-effects meta-regression using baseline LVEF as a continuous moderator for both outcomes (29). We used Egger's regression test to examine publication bias when 10 or more reports with the same outcome were available (30). Whenever possible, we planned subgroup analyses based on risk of bias and beta-blocker type performed for all outcomes. Additionally, trial sequential analysis (TSA) was conducted using the Copenhagen Trial Unit TSA software (version 0.9.5.10 Beta) to assess the conclusiveness of statistically significant results and to determine whether inconclusive findings necessitated additional sample size (31). TSA was applied to continuous outcomes, with MD. The Required Information Size (RIS) was estimated based on expected effect sizes and event rates, and when appropriate, an event size-based information axis was used.

## 3 Results

### 3.1 Study selection

Our initial search identified 1,043 potential articles across four databases. After removing 229 duplicate articles, we conducted screening based on title and abstract, leading to the exclusion of 781 articles. Of the 33 articles selected, 1 could not be retrieved. The remaining 32 articles were assessed for eligibility, excluding 8 for wrong population, 7 for wrong intervention, 6 for wrong study design, 2 for wrong outcome, and 1 for foreign language. For this review, we included 8 studies that met the eligibility criteria. We summarized this study selection process in the PRISMA flow diagram (Figure 1).

**Figure 1:**
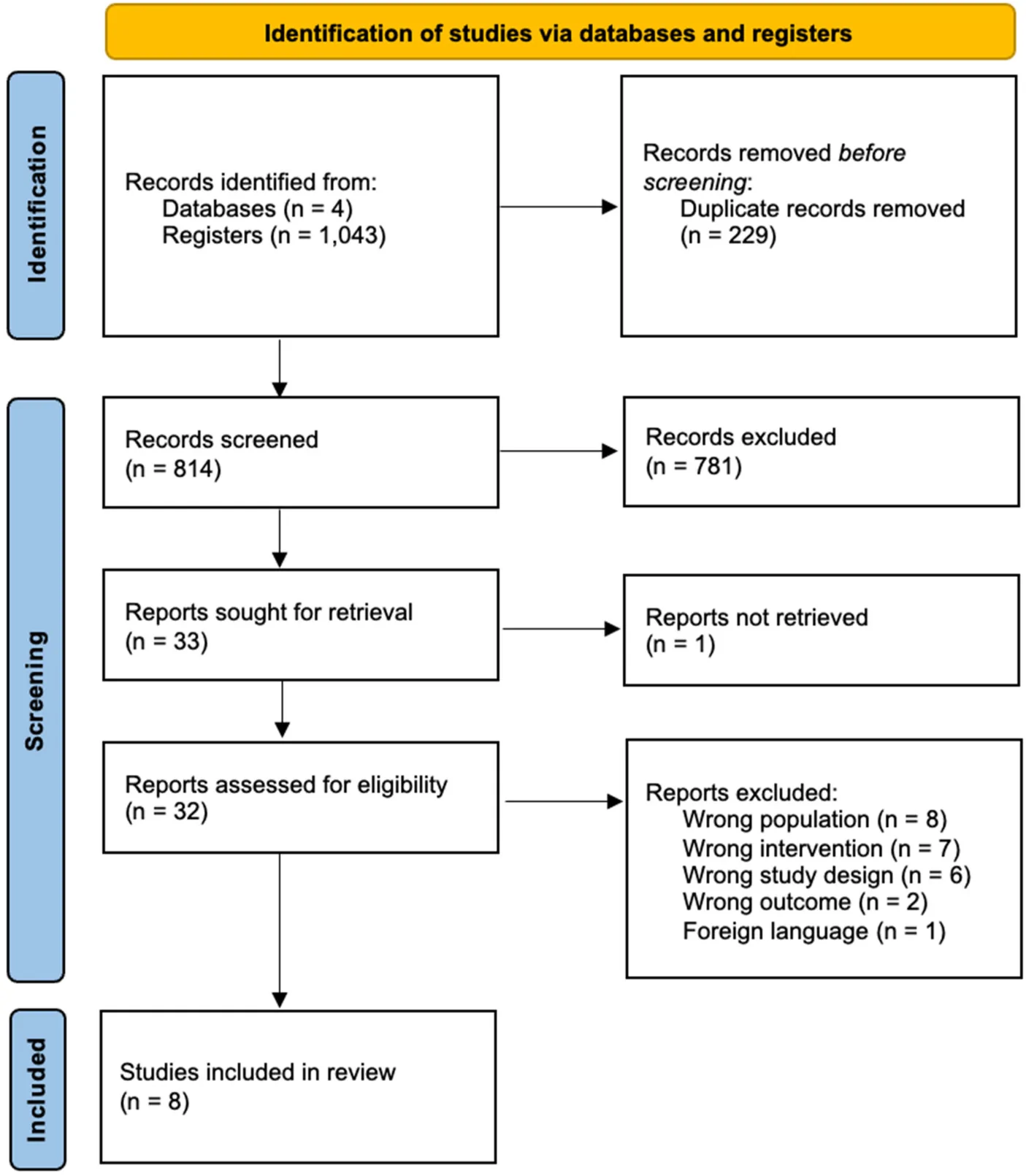
Flow diagram illustrating the study selection process for updated systematic reviews. Initial search across four databases yielded a total of 1,043 records. After removing 229 duplicate records, we conducted screening based on title and abstract, leading to exclusion of 781 articles. Of the 33 articles selected, 1 could not be retrieved. Of the 32 reports assessed for eligibility, 24 studies were excluded based upon specific exclusion criteria, including wrong population (*n* = 8), wrong intervention (*n* = 7), wrong study design (*n* = 6), wrong outcome (*n* = 2), and non-English language (*n* = 1). The remaining 8 studies met the inclusion criteria and were included in the systematic review. This process adheres to the PRISMA guidelines for reporting updated systematic reviews and meta-analysis.

### 3.2 Characteristics of included studies

We evaluated 7 randomized controlled trials (RCTs) and 1 *post-hoc* study. Across all studies, a total of 772 unique participants were enrolled. The studies were geographically diverse, with the largest contribution from Iran (25%), Turkey (25%) Brazil (12.5%), Romania (12.5%), Italy (12.5%), and Korea (12.5%). The follow-up durations ranged from 1 to 24 months.

Regarding LVEF, 50% of studies reported a statistically significant preservation of LVEF favoring beta-blockers compared to placebo/control and 50% showed no differences among groups. For E/A ratio, 50% reported no difference among groups, and 50% did not assess this parameter. We summarized this information in (Table 1) (4–10).

**Table 1:** Summary of included studies.

Author and year	Country	Study design	CT protocol	Treatment comparisons	Total sample Size	Sample size cases	Sample size control	Baseline LVEF mean cases (SD)	Baseline LVEF mean controls (SD)	Key points
Avila et al. (5)	Brazil	RCT	Doxorubicin + cyclophosphamide + paclitaxel	Carvedilol 25 mg every 12 h vs. placebo	192	96	96	64.8 (4.7)	65.2 (3.6)	LVEF was comparable between groups.
Beheshti et al. (6)	Iran	RCT	Doxorubicin + cyclophosphamide + paclitaxel	Carvedilol 6.25 mg twice daily vs. 2 placebo tablets	70	30	40	NA	NA	There was no statistically significant difference in LVEF between groups.
Cochera et al. (4)	Romania	RCT	doxorubicin monotherapy	Nebivolol 5 mg daily vs. standard care	60	30	30	62 (4)	61 (2)	No statistically significant differences were observed among groups in LVEF and E/A ratio.
Elitok et al. (9)	Turkey	RCT	ANT therapy	Carvedilol 12.5 mg daily vs. standard care	80	40	40	65 (4.5)	66 (6.1)	No statistically significant differences were observed among groups in LVEF and E/A ratio.
Kaya et al. (8)	Turkey	RCT	Cyclophosphamide + epirubicin + 5-FU OR adriamycin + cyclophosphamide	Nebivolol 5 mg daily vs. placebo	45	27	18	65.6 (4.8)	66.6 (5.5)	Nebivolol was associated with a statistically significant increase in LVEF and E/A ratio compared with placebo.
Lee et al. (38)	Korea	Post-hoc	Surgery + neoadjuvant or adjuvant doxorubicin	Carvedilol 3.125 mg daily	113	70	43	64.6 (2.3)	64.5 (2.9)	Carvedilol showed a statistically significant higher LVEF% than control
Meattini et al. (7)	Italy	RCT	AC-based regimen	Bisoprolol 5 mg vs. placebo	131	66	65	65.8 (1.4)	64 (1.5)	Bisoprolol showed a statistically significant increase in LVEF compared with placebo.
Nabati et al. (10)	Iran	RCT	doxorubicin + cyclophosphamide	Carvedilol 6.125mg twice daily vs. standard care	81	41	40	58.7 (4.6)	61.1 (4.9)	Carvedilol showed a statistically significant increase in LVEF, but no difference in E/A ratio compared with control.

LVEF, left ventricular ejection fraction; SD, standard deviation; CT, Chemotherapy; RCT, randomized control trial; E/A, early to atrial ventricular filling velocity ratio.

### 3.3 Risk of bias

We evaluated the risk of bias in 7 RCTs and 1 *post-hoc* study using the RoB 2.0 tool (20). Of these, three (37.5%) studies had some concerns, and five (62.5%) were considered low risk (Figure 2). Based on these assessments, 62.5% of the studies were considered high quality, and 37.5% moderate quality.

**Figure 2:**
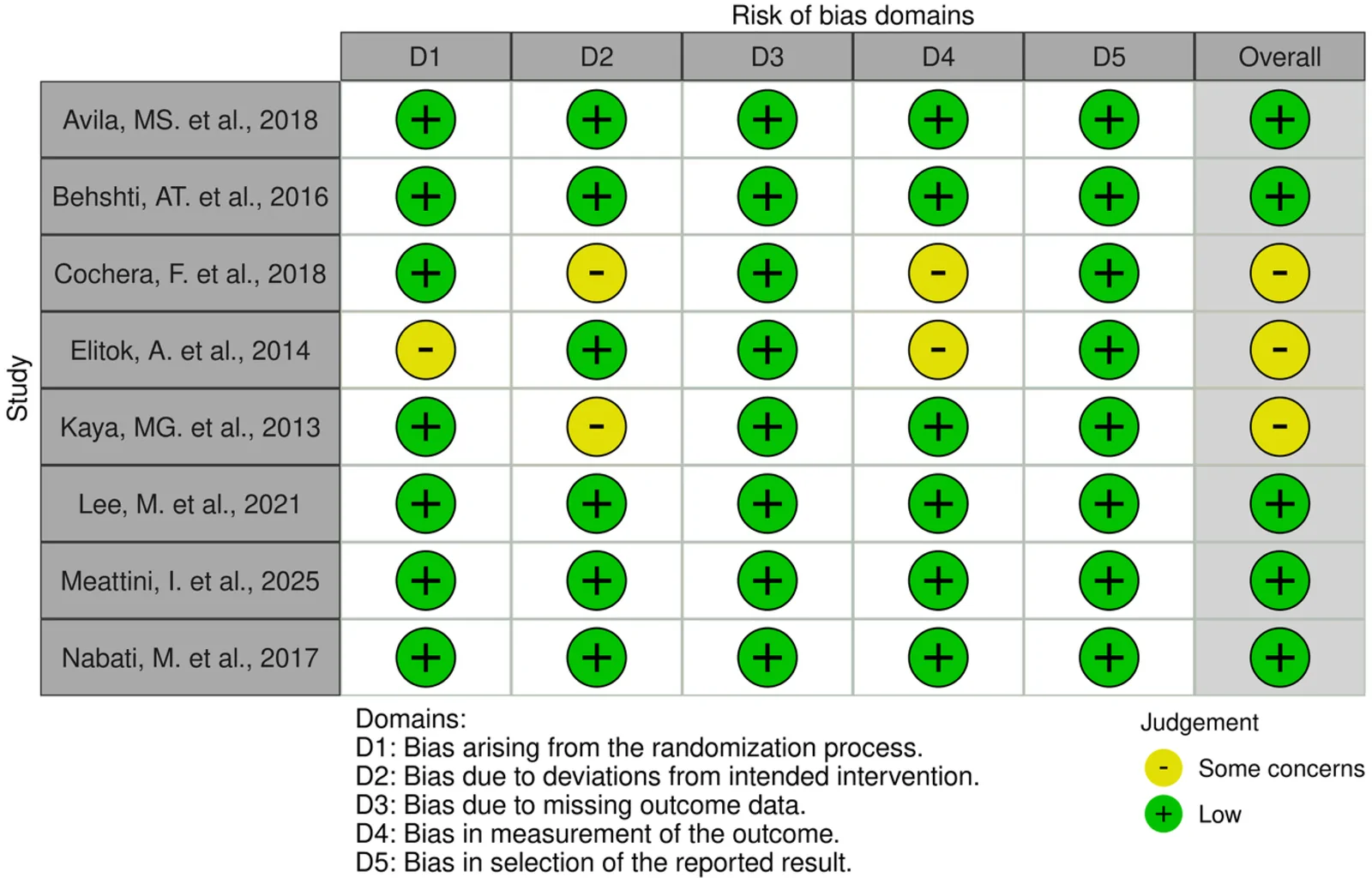
Risk of bias assessment of randomized trials (RoB 2.0 tool). This figure represents a risk of bias summary table evaluating eight studies across five bias domains (D1–D5). The domains included randomization, intervention deviations, missing data, outcome measurements, and reported result selection. The color-coded system-green for low risk, yellow for some concerns, and red for red risk-visually conveys the risk in each domain and overall, per study. Five studies showed a low risk of bias and three studies showed some concerns**.**

### 3.4 Meta-analysis

#### 3.4.1 LVEF

In this meta-analysis, we included 8 studies with a total sample size of 772 participants, of whom 400 received beta-blockers and 372 were assigned to the control group. Using a random-effects model, the pooled MD was estimated at 2.85 (95% CI: 0.58 to 5.12, *p* = 0.02; *I*^2^ = 95%, Figure 3A). The funnel plot revealed asymmetry suggestive of potential publication bias (Supplementary Figure 1A). The Egger regression test was not conducted due to a low number of studies. Subgroup analysis demonstrated significant differences according to beta-blocker type (*p* < 0.01), whereas no significant differences were observed across risk of bias subgroups (*p* = 0.80; Supplementary Table 5). Influence analysis identified Meattini et al. as influential study; its exclusion reduced both the pooled effect (MD = 2.10, 95% CI: 0.62 to 3.58, *p* < 0.01) and heterogeneity (*I*^2^ = 75.2%), although the effect remained statistically significant (Supplementary Table 6). Random-effect meta-regression using baseline LVEF as a moderator showed no significant association with the treatment effect (Supplementary Figure 2A). The TSA estimated a RIS of 1,785 patients. Although the Z-curve crossed the monitoring boundaries in favor of beta-blockers, the RIS was not reached (Figure 4A).

**Figure 3:**
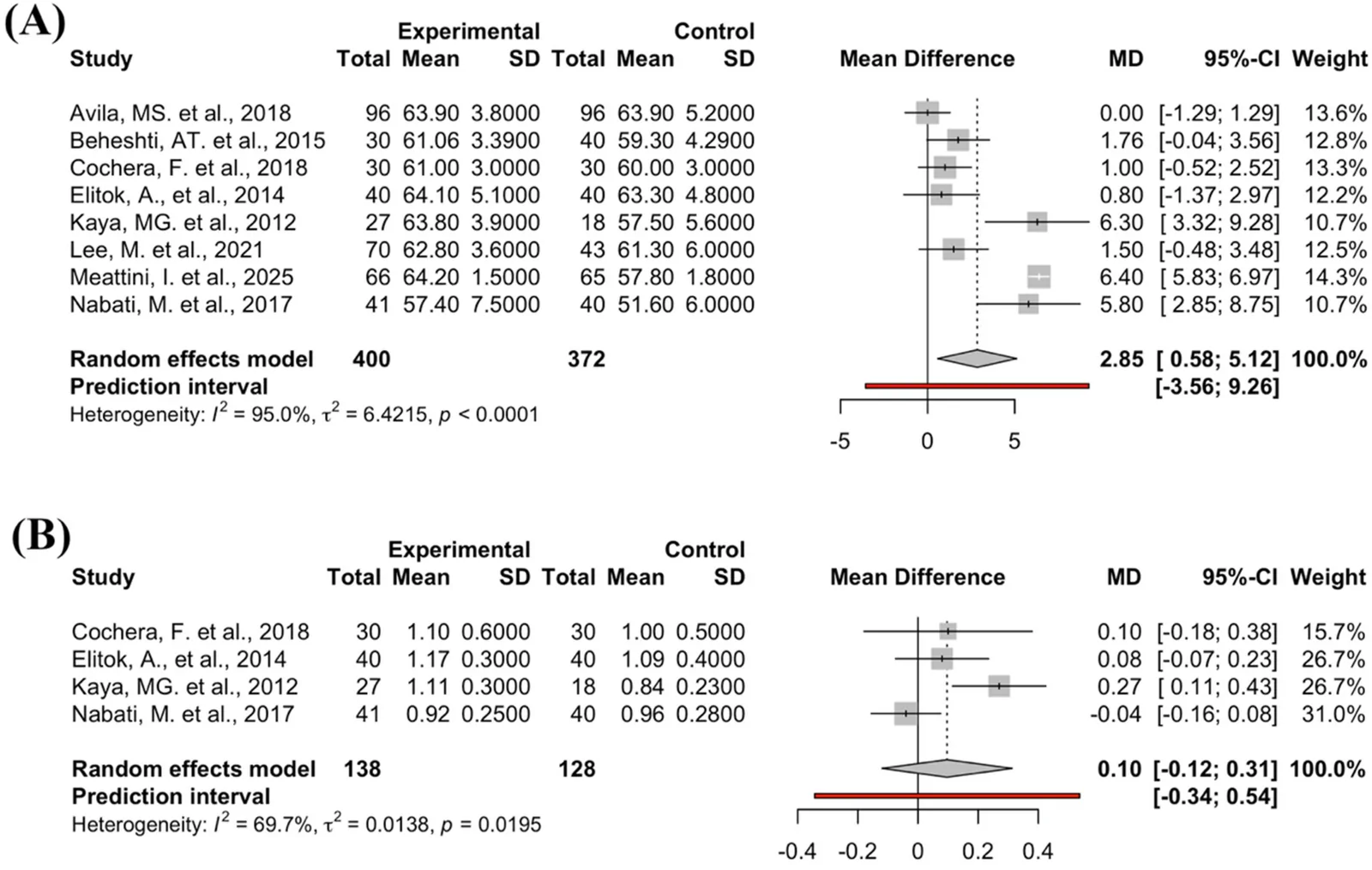
Forest plots of echocardiographic outcomes comparing beta-blockers vs. control. **(A)** Left ventricular ejection fraction (LVEF); **(B)** E/A ratio. Squares represent study-specific effect estimates, with size proportional to study weight; diamonds indicate pooled estimates under a random-effects model, and horizontal lines denote 95% confidence intervals.

**Figure 4:**
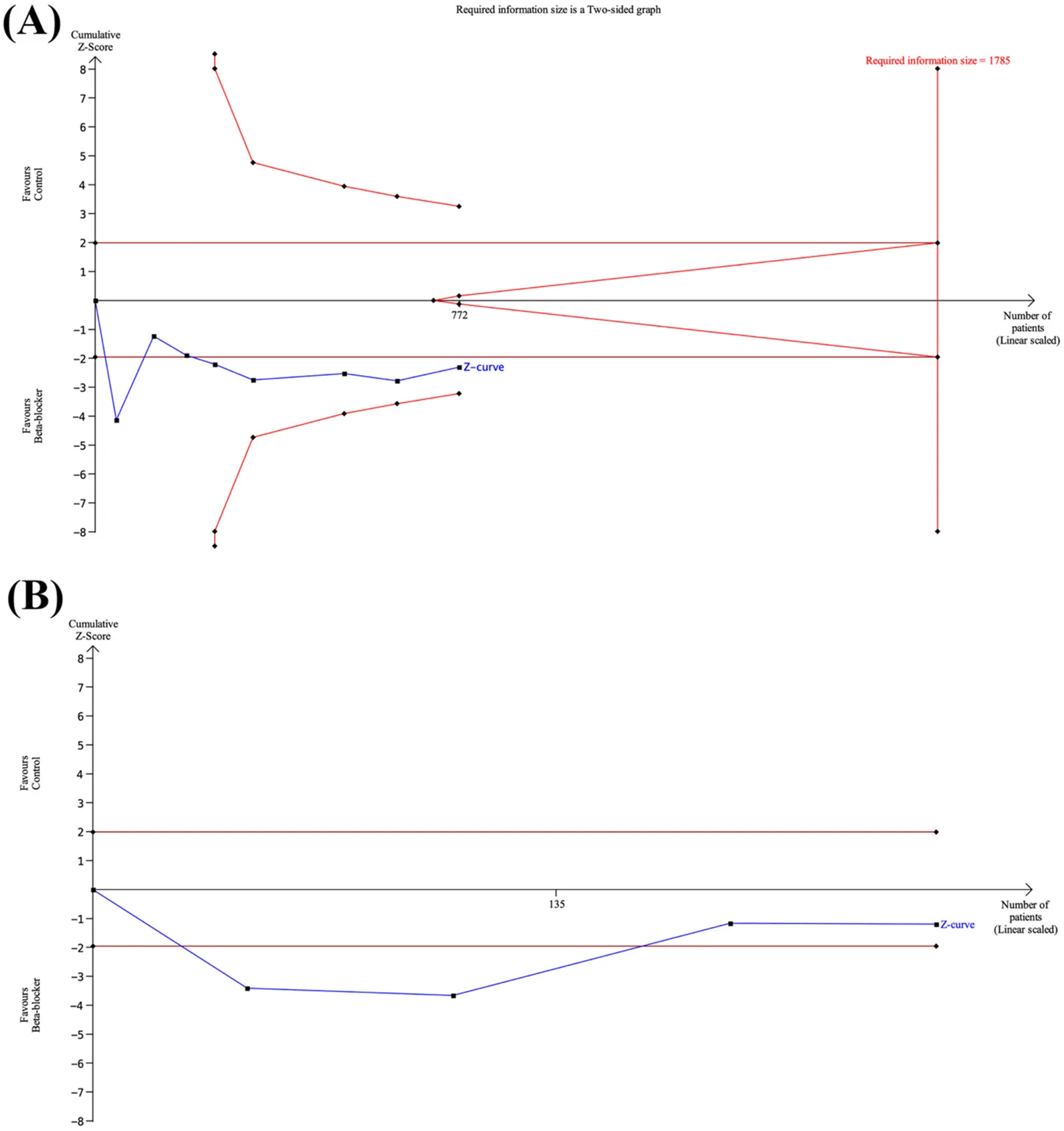
Trial sequential analyses of echocardiographic outcomes comparing beta-blockers vs. control. **(A)** Left ventricular ejection fraction (LVEF); **(B)** E/A ratio. In each panel, the blue line represents the cumulative Z-curve as studies are added sequentially. The inner horizontal red lines indicate the conventional boundaries for statistical significance (*Z* = ±1.96), and the outer curved red lines represent the trial sequential monitoring boundaries for benefit and harm. The vertical red line denotes the required information size (RIS). Crossing a trial sequential monitoring boundary suggests that the evidence may be sufficient to support a conclusive result, whereas failure to cross these boundaries indicates that additional studies may be needed.

#### 3.4.2 E/A ratio

In this meta-analysis, we included 4 studies with a total sample size of 266 participants, including 138 individuals treated with beta-blockers and 128 assigned to the control group. Using a random-effects model, the pooled MD was estimated at 0.10 (95% CI: −0.12 to 0.31, *p* = 0.25; *I*^2^ = 69.7%, Figure 3B). The funnel plot demonstrated relative symmetry, suggesting no major publication bias (Supplementary Figure 1B). The Egger regression test was not conducted due to a low number of studies. Subgroup analysis demonstrated a statistically significant difference according to risk of bias (*p* = 0.02) and beta-blocker type (*p* = 0.02), with reduced heterogeneity observed (Carvedilol *I*^2^ = 32.4% and Nebivolol *I*^2^ = 7.9%; Supplementary Table 7). Influence analysis identified two potentially influential studies (Kaya et al. and Nabati et al.); their exclusion resulted in a marked reduction in heterogeneity (*I*^2^ = 0% and 35.9%, respectively). Exclusion of Nabati et al. resulted in a statistically significantly pooled effect (MD = 0.16, 95% CI: 0.03 to 0.29, *p* = 0.01) (Supplementary Table 8). Meta-regression using baseline LVEF as a moderator showed a significant positive association with the treatment effect on E/A ratio (*β* = 0.03, 95% CI: 0.002 to 0.06, *p* = 0.04, *R*^2^ = 74.7%) (Supplementary Figure 2B). Given the limited number of studies, this finding should be interpreted as exploratory. The TSA did not estimate RIS because of insufficient available information. The Z-curve remained withing the monitoring boundaries (Figure 4B).

### 3.5 Assessment of the certainty of the evidence and summary of findings

According to the GRADE assessment, the certainty of evidence was very low for LVEF and low for E/A ratio (Supplementary Table 9). Downgrading was primarily driven by substantial heterogeneity, imprecision of effect estimates, and potential publication bias for the LVEF outcome. This suggests that although beta-blockers may preserve cardiac function, confidence in the magnitude and consistency of the observed effects is limited, highlighting the need for larger and higher-quality randomized trials.

## 4 Discussion

In this GRADE-assessed systematic review and meta-analysis of eight studies comprising 772 patients with breast cancer receiving anthracycline-based chemotherapy, beta-blocker therapy was associated with statistically significant preservation of LVEF compared with placebo or standard care (MD 2.85 percentage points; 95% CI, 0.58–5.12; *p* = 0.02). This finding is biologically plausible: anthracyclines injure cardiomyocytes through reactive oxygen species generation, topoisomerase II-*β* inhibition, mitochondrial dysfunction, and apoptotic cascade activation, which collectively trigger sympathoadrenal axis stimulation and maladaptive ventricular remodeling (4–10). Beta-blockers interrupt this cascade by attenuating catecholamine-mediated beta-1 adrenergic receptor activation, thereby reducing calcium overload, oxidative stress, and cardiomyocyte apoptosis, with carvedilol and nebivolol conferring additional antioxidant and nitric oxide–mediated protection beyond pure sympatholysis (32, 33). This benefit should be interpreted in the context of substantial heterogeneity. Subgroup analysis demonstrated significant differences according to beta-blocker type, suggesting that cardioprotective effects may vary across individual agents; however, given the limited number of studies evaluating each beta-blocker, these findings should be considered exploratory (4, 6, 8–10). Differences in dose and follow-up duration across trials may have also contributed to between-study variability (4–10). TSA supported the potential benefit of beta-blockers, as the Z-curve crossed the monitoring boundary; however, the RIS was not reached, indicating the current evidence remains underpowered for definitive conclusions.

The potential cardioprotective effects of beta-blockers should also be interpreted in the context of their distinct pharmacologic properties rather than as a uniform class effect. Among these agents, carvedilol combines nonselective *β*1/*β*2-adrenergic blockade with *α*1-adrenergic antagonism, displays vasodilating properties, and has potent antioxidant effects that may be particularly relevant to anthracycline-induced oxidative and mitochondrial injury (33). Its antioxidant activity, including free-iron chelation, may further reduce doxorubicin-mediated free-radical toxicity and lipid peroxidation (33). Nebivolol, in contrast, is a highly *β*1-selective agent that promotes nitric oxide-mediated vasodilation through *β*3-receptor agonism and stimulation of endothelial nitric oxide synthase, a mechanism distinct from the *α*-adrenergic blockade underlying carvedilol-associated vasodilation (34). Bisoprolol is likewise predominantly *β*1-selective and exerts cardioprotective effects through adrenergic blockade. Although it attenuated treatment-related LVEF decline in MANTICORE 101-Breast without preventing LV remodeling, it also prevented worsening of peak GLS in the SAFE trial; unlike carvedilol, however, it does not appear to prevent redox-dependent *β*1-adrenergic receptor inactivation (32, 35). Consistent with these pharmacologic differences, our subgroup analyses demonstrated significant variation in pooled effects according to beta-blocker type. However, the small number of studies evaluating each individual agent precludes reliable conclusions regarding comparative efficacy, and these findings should therefore be considered hypothesis-generating rather than evidence of superiority of one beta-blocker over another.

The magnitude of LVEF preservation (2.85 percentage points) warrants contextual interpretation. Contemporary guidelines define clinically meaningful cardiotoxicity as a ≥10 percentage point of absolute LVEF decline or a drop below 53%. While our pooled estimate falls below this threshold at the group level, ceiling effects from high baseline LVEF values across trials (62%–66%) and population-level averaging may underestimate benefit in higher-risk subpopulations. To further explore this potential source of variability, we performed a meta-regression using baseline LVEF as a moderator; however, there was no association with the treatment effect for LVEF. From a long-term survivorship perspective, where cardiovascular mortality increasingly competes with cancer-specific mortality, even modest attenuation of subclinical systolic decline may carry meaningful downstream implications (2, 3).

Our findings are consistent with, and add mechanistic precision to, the broader cardioprotection literature. Lewinter et al. reported a significant beta-blocker effect on LVEF across mixed anthracycline and trastuzumab populations (MD, 2.4%; *I*^2^ = 82%), though the anthracycline-specific subgroup alone was non-significant (MD, 1.9%; 95% CI, −0.5 to 4.2) (11). Gao et al. similarly demonstrated LVEF preservation across 13 RCTs combining both treatments (SMD, 0.393; *I*^2^ = 70.8%) (12). By primarily focusing on anthracycline-treated populations and isolating beta-blocker-based strategies, our analysis provides a more focused evaluation of beta-blocker cardioprotection. Li et al., the most recent comparable meta-analysis, reported a directionally consistent but non-significant LVEF effect (MD, 2.73%; 95% CI, −0.45 to 5.92; very low QoE), suggesting that population heterogeneity from mixed-drug designs attenuates statistical precision (36). The 2025 SAFE trial reinforces the plausibility of beta-blocker cardioprotection in this setting. Although the SAFE trial used a factorial design including bisoprolol and ramipril, only the beta-blocker intervention arm was incorporated into the present analysis (7).

The relatively modest number of eligible studies therefore reflects the intentionally focused clinical question rather than an incomplete assessment of the available literature. Broadening eligibility to include trastuzumab-only populations, non-randomized clinical trials, other malignancies, or cardioprotective regimens combining beta-blockers with additional neurohormonal agents would increase the nominal sample size but would also introduce important clinical and mechanistic heterogeneity and reduce the ability to isolate beta-blocker-associated effects. Accordingly, we favored a more homogeneous anthracycline-treated breast cancer population while recognizing that this methodological choice necessarily limits statistical power and the precision of agent-specific estimates.

In contrast, beta-blocker therapy was not associated with a statistically significant improvement in E/A ratio (MD, 0.10; 95% CI, −0.12 to 0.31; *p* = 0.25). Mechanistically, diastolic dysfunction in anthracycline cardiotoxicity arises from impaired sarcoplasmic reticulum calcium reuptake, titin oxidative modification, and myocardial fibrosis, pathways less directly targeted by sympatholytic beta-blockade, while the E/A ratio itself is a load-dependent and relatively insensitive marker of early diastolic change (37). Although the overall effect on E/A ratio was not significant, sensitivity analysis showed that exclusion of Nabati et al. resulted in a significant benefit with reduced heterogeneity, whereas exclusion of Kaya et al. reduced heterogeneity to 0% (8, 10). Meta-regression also demonstrated a significant association between baseline LVEF and the treatment effect on E/A ratio. TSA remained inconclusive because the available information was insufficient to estimate the required information size. Given that only four studies contributed to this outcome, these findings should be interpreted as exploratory.

The certainty of evidence was very low for LVEF and low for E/A ratio, driven by heterogeneity, imprecision, and possible publication bias, consistent with ratings from Lewinter et al. and Li et al. (11, 36). Overall, beta-blockers may attenuate anthracycline-related systolic dysfunction in patients with breast cancer, but the limited number of available trials, substantial heterogeneity, and low certainty of evidence preclude definitive conclusions regarding either the magnitude of cardioprotection or the relative efficacy of individual agents. Future adequately powered trials should incorporate agent-specific treatment arms, standardized beta-blocker dosing, longer follow-up, clinically meaningful cardiovascular outcomes, and contemporary multiparametric cardiac imaging to determine whether the mechanistic differences among carvedilol, nebivolol, and bisoprolol translate into clinically relevant differences in cardioprotection.

## 5 Strengths and limitations

This study has several important strengths. Unlike prior meta-analyses evaluating multiple cardioprotective strategies simultaneously, this analysis specifically focused on beta-blocker-based interventions, allowing a more mechanistically focused assessment of the independent cardioprotective role of beta-blockade in predominantly anthracycline-treated breast cancer populations. The evidence base was predominantly derived from randomized controlled trials, enhancing internal validity and reducing the risk of confounding. Additionally, this analysis incorporated contemporary evidence, including the recently published SAFE trial (7), and applied rigorous methodological approaches, including GRADE assessment and TSA.

However, several limitations should be acknowledged. Most importantly, despite an updated systematic search, only eight eligible studies comprising 772 participants were identified, reflecting the limited randomized evidence available for this narrowly defined clinical question. This relatively small evidence base reduces the precision of pooled estimates and substantially limits the reliability of comparisons among individual beta-blockers. Accordingly, the significant differences observed across beta-blocker subgroups should be regarded as exploratory and should not be interpreted as establishing superiority of carvedilol, nebivolol, or bisoprolol. A major additional limitation was the substantial heterogeneity observed for LVEF outcomes, likely reflecting variability in beta-blocker type, dose, cumulative anthracycline exposure and follow-up duration across included trials. Additionally, some included studies permitted concomitant trastuzumab exposure, which may have introduced residual mechanistic heterogeneity. In addition, follow-up durations were relatively short in several studies, potentially limiting assessment of delayed cardiotoxicity. Most included trials primarily relied on echocardiographic surrogate markers rather than clinical cardiovascular outcomes such as symptomatic heart failure, cardiovascular mortality, or hospitalization. Furthermore, the certainty of evidence ranged from low to very low because of heterogeneity, imprecision, and possible publication bias.

Future research should focus on adequately powered randomized controlled trials with longer follow-up durations, standardized cardiotoxicity definitions, and clinically meaningful cardiovascular outcomes. Additional studies are needed to clarify whether cardioprotective efficacy differs according to beta-blocker type and cumulative anthracycline exposure. Incorporation of contemporary imaging modalities may further define the role of beta-blockers as preventive cardio-oncology strategies in patients receiving anthracycline-based chemotherapy.

## 6 Conclusion

In this systematic review and meta-analysis of randomized evidence involving patients with breast cancer receiving anthracycline-based chemotherapy, beta-blocker therapy was associated with statistically significant preservation of LVEF, suggesting a potential cardioprotective effect against anthracycline-related systolic dysfunction. TSA supported the direction of benefit; however, the RIS was not reached, and substantial heterogeneity remained across studies. In contrast, no significant benefit was observed for E/A ratio, and evidence regarding diastolic protection remains inconclusive. Overall, the certainty of evidence ranged from low to very low, highlighting the need for larger, adequately powered randomized trials with longer follow-up durations and clinically meaningful cardiovascular outcomes to better define the role of beta-blockers as preventive cardio-oncology strategies in anthracycline-treated breast cancer patients.

## Data Availability

All the data used to perform this analysis is publicly available in the referenced articles. The rest of the material is at the author's disposition upon reasonable request. Requests to access the datasets should be directed to lorenamurrieta@hotmail.com.
