# Supplementary material for: Efficacy of beta-blockers in preventing cardiotoxicity in breast cancer patients receiving anthracyclines: a GRADE-assessed systematic review and meta-analysis with trial sequential analysis
Source: Front Cardiovasc Med. 2026 Oct 9;13:1926758. doi: 10.3389/fcvm.2026.1926758 (PMC13690626; doi:10.3389/fcvm.2026.1926758)
Supplement: Supplementary file 1 [file Datasheet1.docx]

**Efficacy of Beta-Blockers in Preventing Cardiotoxicity in Breast Cancer Patients Receiving Anthracyclines: A GRADE-Assessed Systematic Review and Meta-Analysis with Trial Sequential Analysis**

Victor M Samperio, MD^1^; Lorena M. Murrieta Bruciaga, MD^2^; Jesús Antonio Castañeda Martínez, MD^3^; Vanessa Coronado Porchas, MD^4^; Alejandro Chen Liang, MD^4^; Yeison Cruz Castillo, MD^5^; Aishwarya Ghonge, MD^1^; Aishwarya Saripalli, MD^6^; Thomas Schulz, MD^7^

^1^Department of Medicine, Eisenhower Medical Center, Rancho Mirage, CA, USA

^2^Universidad del Valle de México, Hermosillo, Sonora, México

^3^Universidad de Sonora, Hermosillo, Sonora, México

^4^Centro de Estudios Universitarios Xochicalco Campus Tijuana, Tijuana, Baja California, México

^5^Universidad Autónoma de Santo Domingo, Santo Domingo, República Dominicana

^6^ Kern Medical Center, Bakersfield, CA

^7^Lucy Curci Cancer Center, Eisenhower Health, Rancho Mirage, CA, USA

^1^ [samperio.vm@gmail.com](mailto:samperio.vm@gmail.com) | [^2^](mailto:2samperio.vm@gmail.com)[lorenamurrieta@hotmail.com](mailto:lorenamurrieta@hotmail.com) ^3^[Jesus.anthony.cm@gmail.com](mailto:Jesus.anthony.cm@gmail.com) | ^4^[vanessa_coro98@hotmail.com](mailto:vanessa_coro98@hotmail.com) ^4^[alex95chenliang@gmail.com](mailto:alex95chenliang@gmail.com) | ^5^[yeisoncc_159@hotmail.com](mailto:4yeisoncc_159@hotmail.com) | ^1^[aishwaryaghonge@gmail.com](mailto:6aishwaryaghonge@gmail.com) | ^6^[aishwarya.saripalli94@gmail.com](mailto:aishwarya.saripalli94@gmail.com) | ^7^[tschulz@eisenhowerhealth.org](mailto:tschulz@eisenhowerhealth.org) |

**ORCID:** ^1^[0000-0003-3445-7546](https://orcid.org/0000-0003-3445-7546) | ^2^0009-0005-1317-7379 | ^3^0000-0002-8419-8194 | ^4^0009-0009-5075-3821 | ^4^0009-0005-6769-7505 | ^5^0009-0005-3660-1304 | ^6^0000-0002-0327-6163

**Corresponding Authors:**

Victor M Samperio, MD ([samperio.vm@gmail.com](mailto:samperio.vm@gmail.com))

Lorena M. Murrieta Bruciaga, MD ([lorenamurrieta@hotmail.com](mailto:lorenamurrieta@hotmail.com))

Table of contents

[Supplementary table 1. Search in PubMed	3](#_Toc238664968)

[Supplementary table 2. Search in Web of Science	3](#_Toc238664969)

[Supplementary table 3. Search in CINAHL	4](#_Toc238664970)

[Supplementary table 4. Search in Cochrane	5](#_Toc238664971)

[Supplementary Table 5. Subgroup Analysis: LVEF	6](#_Toc238664972)

[Supplementary Table 6. Influence Analysis for LVEF	7](#_Toc238664973)

[Supplementary Table 7. Subgroup Analysis: E/A ratio	7](#_Toc238664974)

[Supplementary Table 8. Influence Analysis for EA	8](#_Toc238664975)

[Supplementary Table 9. GRADE assessement	8](#_Toc238664976)

[Supplementary Figure 1. Funnel plot of the outcomes	9](#_Toc238664977)

[Supplemental Figure 2. Meta-regression of the outcomes	10](#_Toc238664978)

[PRISMA Checklist	11](#_Toc238664979)

[PRISMA Abstract Checklist	14](#_Toc238664980)

# **Supplementary table 1.** Search in PubMed

| Boolean search | Results |
| --- | --- |
| (  "Breast Neoplasms"[Mesh] OR breast cancer OR breast neoplasm* OR mammary carcinoma  )  AND  (  "Anthracyclines"[Mesh] OR anthracycline* OR doxorubicin OR epirubicin OR daunorubicin  )  AND  (  "Adrenergic beta-Antagonists"[Mesh] OR beta-blocker* OR carvedilol OR metoprolol OR bisoprolol OR nebivolol  OR  "Angiotensin-Converting Enzyme Inhibitors"[Mesh] OR ACE inhibitor* OR enalapril OR lisinopril OR ramipril  OR  "Angiotensin Receptor Antagonists"[Mesh] OR ARB OR angiotensin receptor blocker* OR losartan OR valsartan OR candesartan  )  AND  (  cardiotoxicity OR cardiomyopathy OR "heart failure" OR "ventricular dysfunction" OR LVEF OR "ejection fraction" OR GLS OR "global longitudinal strain"  ) | 142 |

# **Supplementary table 2.** Search in Web of Science

| Boolean search | Results |
| --- | --- |
| TS=(  ("breast cancer" OR "breast neoplasm*" OR "mammary carcinoma")  AND  (anthracycline* OR doxorubicin OR epirubicin OR daunorubicin)  AND  (  beta-blocker* OR carvedilol OR metoprolol OR bisoprolol OR nebivolol  OR  "ACE inhibitor*" OR enalapril OR lisinopril OR ramipril  OR  ARB OR "angiotensin receptor blocker*" OR losartan OR valsartan OR candesartan  )  AND  (cardiotoxicity OR cardiomyopathy OR "heart failure" OR LVEF OR "ejection fraction" OR GLS)  ) | 222 |

# **Supplementary table 3.** Search in CINAHL

| Boolean search | Results |
| --- | --- |
| (  (MH "Breast Neoplasms+") OR TI (breast cancer OR breast neoplasm* OR mammary carcinoma)  OR AB (breast cancer OR breast neoplasm* OR mammary carcinoma)  )  AND  (  (MH "Anthracyclines+") OR TI (anthracycline* OR doxorubicin OR epirubicin OR daunorubicin)  OR AB (anthracycline* OR doxorubicin OR epirubicin OR daunorubicin)  )  AND  (  (MH "Adrenergic beta-Antagonists+") OR TI (beta-blocker* OR carvedilol OR metoprolol OR bisoprolol OR nebivolol)  OR AB (beta-blocker* OR carvedilol OR metoprolol OR bisoprolol OR nebivolol)  OR  (MH "Angiotensin-Converting Enzyme Inhibitors+") OR TI (ACE inhibitor* OR enalapril OR lisinopril OR ramipril)  OR AB (ACE inhibitor* OR enalapril OR lisinopril OR ramipril)  OR  (MH "Angiotensin Receptor Antagonists+") OR TI (ARB OR angiotensin receptor blocker* OR losartan OR valsartan OR candesartan)  OR AB (ARB OR angiotensin receptor blocker* OR losartan OR valsartan OR candesartan)  )  AND  (  TI (cardiotoxicity OR cardiomyopathy OR heart failure OR LVEF OR ejection fraction OR GLS)  OR AB (cardiotoxicity OR cardiomyopathy OR heart failure OR LVEF OR ejection fraction OR GLS)  ) | 32 |

# **Supplementary table 4.** Search in Cochrane

| Boolean search | Results |
| --- | --- |
| (  MeSH descriptor: [Breast Neoplasms] explode all trees  )  AND  (  MeSH descriptor: [Anthracyclines] explode all trees OR anthracycline* OR doxorubicin OR epirubicin OR daunorubicin  )  AND  (  MeSH descriptor: [Adrenergic beta-Antagonists] explode all trees OR beta-blocker* OR carvedilol OR metoprolol OR bisoprolol  OR  MeSH descriptor: [Angiotensin-Converting Enzyme Inhibitors] explode all trees OR ACE inhibitor* OR enalapril OR lisinopril OR ramipril  OR  MeSH descriptor: [Angiotensin Receptor Antagonists] explode all trees OR ARB OR losartan OR valsartan OR candesartan  )  AND  (  cardiotoxicity OR cardiomyopathy OR "heart failure" OR LVEF OR "ejection fraction"  ) | 647 |

# **Supplementary Table 5.** Subgroup Analysis: LVEF

| **Variable** | **Subgroup** | **k** | **MD (95% CI)** | **I² (%)** | **p-value (Between Groups)** |
| --- | --- | --- | --- | --- | --- |
| **Beta-blocker type** | Carvedilol | 5 | 1.7052 [-0.8522; 4.2626] | 69.8% | < 0.0001 |
|  | Nebivolol | 2 | 3.4891 [-30.1202; 37.0984] | 89.7% |  |
|  | Bisoprolol | 1 | 6.4000 [ 5.8322; 6.9678] | -- |  |
| **Risk of Bias** | Low | 5 | 3.0537 [-0.4966; 6.6039] | 96.2% | 0.8052 |
|  | Some concerns | 3 | 2.5193 [-5.0041; 10.0427] | 81.2% |  |

# **Supplementary Table 6.** Influence Analysis for LVEF

| **Author** | **Effect (95% CI: Lower; Upper)** | **I²** | **DFFITS** | **Cook’s D** | **QE (del)** | **Is Influential** |
| --- | --- | --- | --- | --- | --- | --- |
| **Avila, MS. et al., 2018** | 3.30 (95% CI: 0.85; 5.74) | 93.3% | -0.478 | 0.213 | 89.847 | no |
| **Beheshti, AT. et al., 2015** | 3.02 (95% CI: 0.34; 5.70) | 95.5% | -0.165 | 0.031 | 132.235 | no |
| **Cochera, F. et al., 2018** | 3.14 (95% CI: 0.53; 5.75) | 95.0% | -0.287 | 0.089 | 119.837 | no |
| **Elitok, A. et al., 2014** | 3.14 (95% CI: 0.56; 5.72) | 95.4% | -0.291 | 0.090 | 130.019 | no |
| **Kaya, MG. et al., 2012** | 2.44 (95% CI: 0.07; 4.81) | 95.7% | 0.440 | 0.183 | 139.646 | no |
| **Lee, M. et al., 2021** | 3.05 (95% CI: 0.40; 5.71) | 95.5% | -0.198 | 0.044 | 132.393 | no |
| **Meattini, I. et al., 2025** | 2.18 (95% CI: -0.03; 4.39) | 75.2% | 0.880 | 0.488 | 24.191 | yes |
| **Nabati, M. et al., 2017** | 2.50 (95% CI: 0.04; 4.96) | 95.7% | 0.370 | 0.135 | 140.354 | no |

# **Supplementary Table 7.** Subgroup Analysis: E/A ratio

| **Variable** | **Subgroup** | **k** | **MD (95% CI)** | **I² (%)** | **p-value (Between Groups)** |
| --- | --- | --- | --- | --- | --- |
| **Beta-blocker type** | Carvedilol | 2 | 0.0085 [-0.7397; 0.7567] | 32.4% | 0.0215 |
|  | Nebivolol | 2 | 0.2264 [-0.7172; 1.1699] | 7.9% |  |
| **Risk of Bias** | Low | 1 | -0.0400 [-0.1557; 0.0757] | -- | 0.0211 |
|  | Some concerns | 3 | 0.1608 [-0.1144; 0.4359] | 35.9% |  |

# **Supplementary Table 8**. Influence Analysis for EA

| **Author** | **Effect (95% CI: Lower; Upper)** | **I²** | **DFFITS** | **Cook’s D** | **QE (del)** | **Is Influential** |
| --- | --- | --- | --- | --- | --- | --- |
| **Cochera, F. et al., 2018** | 0.10 (95% CI: -0.29; 0.49) | 79.72% | -0.01 | 0.00 | 9.86 | no |
| **Elitok, A. et al., 2014** | 0.11 (95% CI: -0.30; 0.52) | 79.77% | -0.10 | 0.01 | 9.89 | no |
| **Kaya, MG. et al., 2012** | 0.02 (95% CI: -0.17; 0.21) | 0.00% | 1.74 | 1.16 | 1.90 | yes |
| **Nabati, M. et al., 2017** | 0.16 (95% CI: -0.11; 0.44) | 35.93% | -1.18 | 0.77 | 3.12 | yes |

# **Supplementary Table 9.** GRADE assessement

| **Certainty assessment** | | | | | | | **№ of patients** | | **Effect** | | **Certainty** | **Importance** |
| --- | --- | --- | --- | --- | --- | --- | --- | --- | --- | --- | --- | --- |
| **№ of studies** | **Study design** | **Risk of bias** | **Inconsistency** | **Indirectness** | **Imprecision** | **Other considerations** | **Beta blockers** | **Control** | **Relative**  **(95% CI)** | **Absolute**  **(95% CI)** |  |  |
| **LVEF%** | | | | | | | | | | | | |
| 8 | randomised trials | not serious | very serious^a^ | not serious | not serious | publication bias strongly suspected^b^ | 400 | 372 | - | MD 2.85 **higher**  (0.58 higher to 5.12 higher) | ⨁◯◯◯  Very low^a,b^ | IMPORTANT |
| **E/A ratio** | | | | | | | | | | | | |
| 4 | randomised trials | not serious | serious^a^ | not serious | serious^c^ | none^b^ | 138 | 128 | - | MD **0.1 higher**  (0.12 lower to 0.31 higher) | ⨁⨁◯◯  Low^a,b,c^ | IMPORTANT |

**CI:** confidence interval; **MD:** mean difference **a**. High heterogeneity **b**. Funnel plot asymmetry **c**. CI crosses null

# **Supplementary Figure 1.** Funnel plot of the outcomes

**Supplemental figure 1.** **A)** LVEF **B)** E/A ratio

# **Supplemental Figure 2.** Meta-regression of the outcomes

**Supplemental figure 2.** **A)** LVEF **B)** E/A ratio

# **PRISMA Checklist**

| **Section and Topic** | **Item #** | **Checklist item** | **Location where item is reported** |
| --- | --- | --- | --- |
| **TITLE** | | |  |
| Title | 1 | Identify the report as a systematic review. | 1 |
| **ABSTRACT** | | |  |
| Abstract | 2 | See the PRISMA 2020 for Abstracts checklist. | --- |
| **INTRODUCTION** | | |  |
| Rationale | 3 | Describe the rationale for the review in the context of existing knowledge. | 5 |
| Objectives | 4 | Provide an explicit statement of the objective(s) or question(s) the review addresses. | 6 |
| **METHODS** | | |  |
| Eligibility criteria | 5 | Specify the inclusion and exclusion criteria for the review and how studies were grouped for the syntheses. | 7 |
| Information sources | 6 | Specify all databases, registers, websites, organisations, reference lists and other sources searched or consulted to identify studies. Specify the date when each source was last searched or consulted. | 8 |
| Search strategy | 7 | Present the full search strategies for all databases, registers and websites, including any filters and limits used. | 8 |
| Selection process | 8 | Specify the methods used to decide whether a study met the inclusion criteria of the review, including how many reviewers screened each record and each report retrieved, whether they worked independently, and if applicable, details of automation tools used in the process. | 8 |
| Data collection process | 9 | Specify the methods used to collect data from reports, including how many reviewers collected data from each report, whether they worked independently, any processes for obtaining or confirming data from study investigators, and if applicable, details of automation tools used in the process. | 9 |
| Data items | 10a | List and define all outcomes for which data were sought. Specify whether all results that were compatible with each outcome domain in each study were sought (e.g. for all measures, time points, analyses), and if not, the methods used to decide which results to collect. | 8 |
|  | 10b | List and define all other variables for which data were sought (e.g. participant and intervention characteristics, funding sources). Describe any assumptions made about any missing or unclear information. | 8 |
| Study risk of bias assessment | 11 | Specify the methods used to assess risk of bias in the included studies, including details of the tool(s) used, how many reviewers assessed each study and whether they worked independently, and if applicable, details of automation tools used in the process. | 9 |
| Effect measures | 12 | Specify for each outcome the effect measure(s) (e.g. risk ratio, mean difference) used in the synthesis or presentation of results. | 10 |
| Synthesis methods | 13a | Describe the processes used to decide which studies were eligible for each synthesis (e.g. tabulating the study intervention characteristics and comparing against the planned groups for each synthesis (item #5)). | 10 |
|  | 13b | Describe any methods required to prepare the data for presentation or synthesis, such as handling of missing summary statistics, or data conversions. | 10 |
|  | 13c | Describe any methods used to tabulate or visually display results of individual studies and syntheses. | 10 |
|  | 13d | Describe any methods used to synthesize results and provide a rationale for the choice(s). If meta-analysis was performed, describe the model(s), method(s) to identify the presence and extent of statistical heterogeneity, and software package(s) used. | 10 |
|  | 13e | Describe any methods used to explore possible causes of heterogeneity among study results (e.g. subgroup analysis, meta-regression). | 10 |
|  | 13f | Describe any sensitivity analyses conducted to assess robustness of the synthesized results. | 10 |
| Reporting bias assessment | 14 | Describe any methods used to assess risk of bias due to missing results in a synthesis (arising from reporting biases). | 10 |
| Certainty assessment | 15 | Describe any methods used to assess certainty (or confidence) in the body of evidence for an outcome. | 10 |
| **RESULTS** | | |  |
| Study selection | 16a | Describe the results of the search and selection process, from the number of records identified in the search to the number of studies included in the review, ideally using a flow diagram. | 11 |
|  | 16b | Cite studies that might appear to meet the inclusion criteria, but which were excluded, and explain why they were excluded. | 11 |
| Study characteristics | 17 | Cite each included study and present its characteristics. | 11 |
| Risk of bias in studies | 18 | Present assessments of risk of bias for each included study. | 12 |
| Results of individual studies | 19 | For all outcomes, present, for each study: (a) summary statistics for each group (where appropriate) and (b) an effect estimate and its precision (e.g. confidence/credible interval), ideally using structured tables or plots. | 12 |
| Results of syntheses | 20a | For each synthesis, briefly summarise the characteristics and risk of bias among contributing studies. | 12 |
|  | 20b | Present results of all statistical syntheses conducted. If meta-analysis was done, present for each the summary estimate and its precision (e.g. confidence/credible interval) and measures of statistical heterogeneity. If comparing groups, describe the direction of the effect. | 12 |
|  | 20c | Present results of all investigations of possible causes of heterogeneity among study results. | 12 |
|  | 20d | Present results of all sensitivity analyses conducted to assess the robustness of the synthesized results. | 12 |
| Reporting biases | 21 | Present assessments of risk of bias due to missing results (arising from reporting biases) for each synthesis assessed. | 12 |
| Certainty of evidence | 22 | Present assessments of certainty (or confidence) in the body of evidence for each outcome assessed. | 12 |
| **DISCUSSION** | | |  |
| Discussion | 23a | Provide a general interpretation of the results in the context of other evidence. | 13 |
|  | 23b | Discuss any limitations of the evidence included in the review. | 15 |
|  | 23c | Discuss any limitations of the review processes used. | 15 |
|  | 23d | Discuss implications of the results for practice, policy, and future research. | 16 |
| **OTHER INFORMATION** | | |  |
| Registration and protocol | 24a | Provide registration information for the review, including register name and registration number, or state that the review was not registered. | 6 |
|  | 24b | Indicate where the review protocol can be accessed, or state that a protocol was not prepared. | 6 |
|  | 24c | Describe and explain any amendments to information provided at registration or in the protocol. | 6 |
| Support | 25 | Describe sources of financial or non-financial support for the review, and the role of the funders or sponsors in the review. | 23 |
| Competing interests | 26 | Declare any competing interests of review authors. | 23 |
| Availability of data, code and other materials | 27 | Report which of the following are publicly available and where they can be found: template data collection forms; data extracted from included studies; data used for all analyses; analytic code; any other materials used in the review. | 23 |

# **PRISMA Abstract Checklist**

| **Section and Topic** | **Item #** | **Checklist item** | **Reported (Yes/No)** |
| --- | --- | --- | --- |
| **TITLE** | | |  |
| Title | 1 | Identify the report as a systematic review. | Yes |
| **BACKGROUND** | | |  |
| Objectives | 2 | Provide an explicit statement of the main objective(s) or question(s) the review addresses. | Yes |
| **METHODS** | | |  |
| Eligibility criteria | 3 | Specify the inclusion and exclusion criteria for the review. | Yes |
| Information sources | 4 | Specify the information sources (e.g. databases, registers) used to identify studies and the date when each was last searched. | Yes |
| Risk of bias | 5 | Specify the methods used to assess risk of bias in the included studies. | Yes |
| Synthesis of results | 6 | Specify the methods used to present and synthesise results. | Yes |
| **RESULTS** | | |  |
| Included studies | 7 | Give the total number of included studies and participants and summarise relevant characteristics of studies. | Yes |
| Synthesis of results | 8 | Present results for main outcomes, preferably indicating the number of included studies and participants for each. If meta-analysis was done, report the summary estimate and confidence/credible interval. If comparing groups, indicate the direction of the effect (i.e. which group is favoured). | Yes |
| **DISCUSSION** | | |  |
| Limitations of evidence | 9 | Provide a brief summary of the limitations of the evidence included in the review (e.g. study risk of bias, inconsistency and imprecision). | Yes |
| Interpretation | 10 | Provide a general interpretation of the results and important implications. | Yes |
| **OTHER** | | |  |
| Funding | 11 | Specify the primary source of funding for the review. | NA |
| Registration | 12 | Provide the register name and registration number. | Yes |
